# Supplementary material for: Transcriptomics and Metabolomics Analyses Reveal High Induction of the Phenolamide Pathway in Tomato Plants Attacked by the Leafminer Tuta absoluta
Source: Metabolites. 2022 May 26;12(6):484. doi: 10.3390/metabo12060484 (PMC9230075; doi:10.3390/metabo12060484)
Supplement: Supplementary file 1 [file metabolites-12-00484-s001.zip › Supplementary data list revised 25 mai.pdf]

Supplementary data files :

- **Supplementary Data S1** : Impact of the *T. absoluta* herbivory on the metabolome of tomato vegetative organs after 7 days of herbivory

- **Supplementary Data S2** : Full-list of identified metabolites which accumulation is impacted y the herbivory of *T. absoluta. This Supplementary Data is given as a Excell file*.

- **Supplementary Data S3** : RNaseq informations

- **Supplementary Data S4** : List of Up and Down DEGs after 5h and 24h of *T. absoluta* herbivory. *This Supplementary Data is given as a Excell file*

- **Supplementary Data S5** : Full gene ontology (GO) terms enrichment at p<0.05 for Up and Down DEGs after 5h and 24h of herbivory. *This Supplementary Data is given as a Excell file*

- **Supplementary Data S6** : KEGG enrichment at $p<0.05$ for Up and Down DEGs after 5h and 24h of herbivory

- **Supplementary Data S7** : List of genes related to polyamine and phenylpropanoid pathway which expression level was followed. *This Supplementary Data is given as a Excell file*

- **Supplementary Data S8** : Medium composition for the NFT tomato culture

- **Supplementary Data S9** : Analytic workflow parameters in Compound Discoverer 3.3

- **Supplementary Data S10** : List of genes and primers

Supplementary Data S1 : Impact of the *T. absoluta* herbivory on the metabolome of tomato vegetative organs after 7 days of herbivory.

A: Venn Diagram representing the number of features significantly affected by the organ nature, *T. absoluta* infestation and their interaction after 7 days of herbivory, determined by a two-way ANOVA (p<0.05). B: Heatmap plot and Hierarchical clustering based on the feature intensities from the five vegetative parts of tomato plant submitted or not to *T. absoluta* herbivory for 7 days. The abbreviations for the organ are the same as in figure 1.

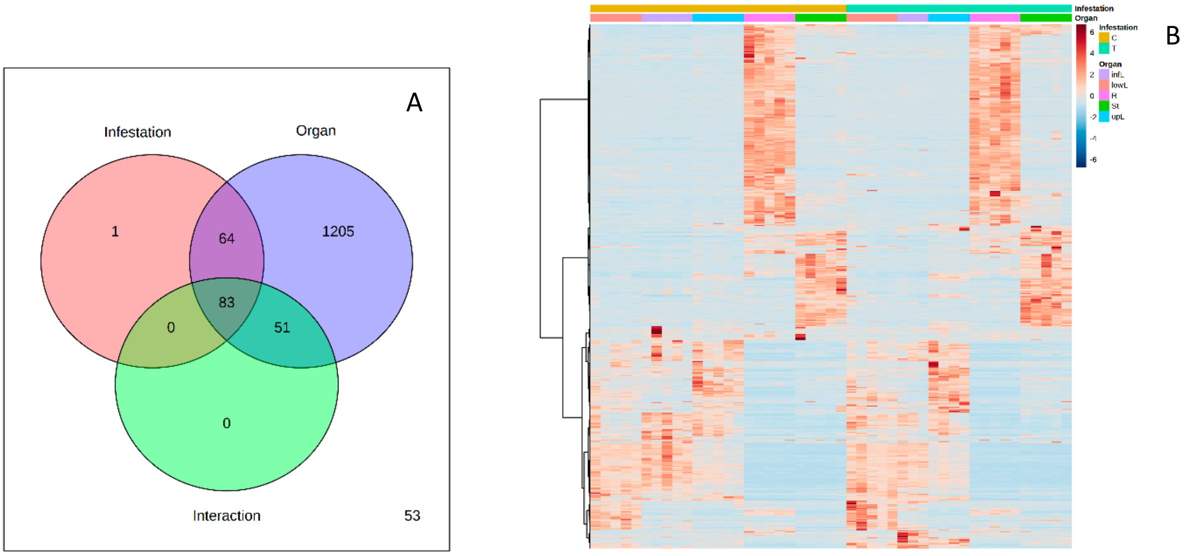

Supplementary data S3 : RNAseq informations.

A: Number of sequenced reads before and after quality control and mapping statistics. B: Multidimensionnal scale plot

A:

	Reads before quality control (trimmomatic)	Reads after quality control (trimmomatic)	Mapped reads	Unmapped reads	%Mapped reads	%Unmapped reads
Tuta_5h_rep1	30799584	29582722	27247219	2335503	92	8
Tuta_5h_rep2	34673862	30913021	28209033	2703988	91	9
Tuta_5h_rep3	29916480	27322327	19655669	7666658	72	28
Tuta_24hrep1	26913288	25381641	22754433	2627208	90	10
Tuta_24hrep2	28420432	27427978	25128775	2299203	92	8
Tuta_24hrep3	30519356	29106302	26745881	2360421	92	8
CTL_5h_rep1	29982924	28574119	26448690	2125429	93	7
CTL_5h_rep2	30584398	28549467	26422174	2127293	93	7
CTL_5h_rep3	31002144	29589775	27374633	2215142	93	7
CTL_24h_rep1	31668134	30051391	27824673	2226718	93	7
CTL_24h_rep2	27906630	26831269	24842498	1988771	93	7
CTL_24h_rep3	26411586	24843117	22945571	1897546	92	8

B:

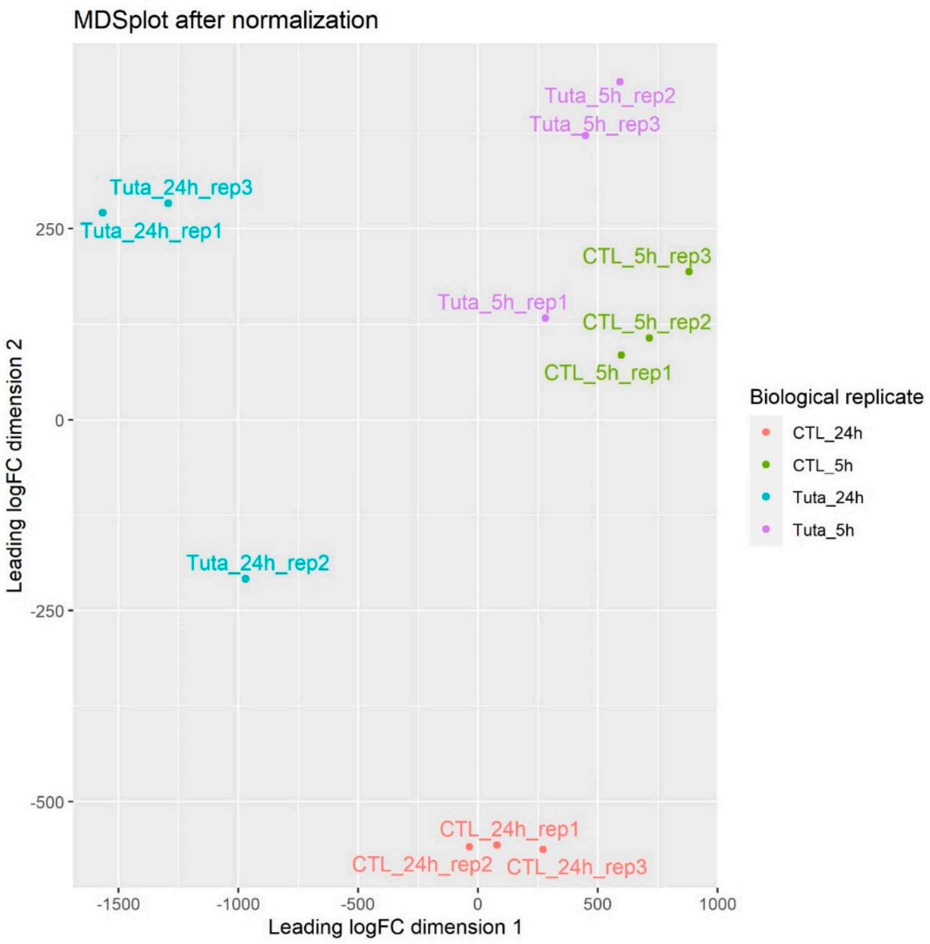

Supplementary data S6 : Kegg enrichment at p<0.05 for up- and down-regulated genes after 5h and 24h of herbivory

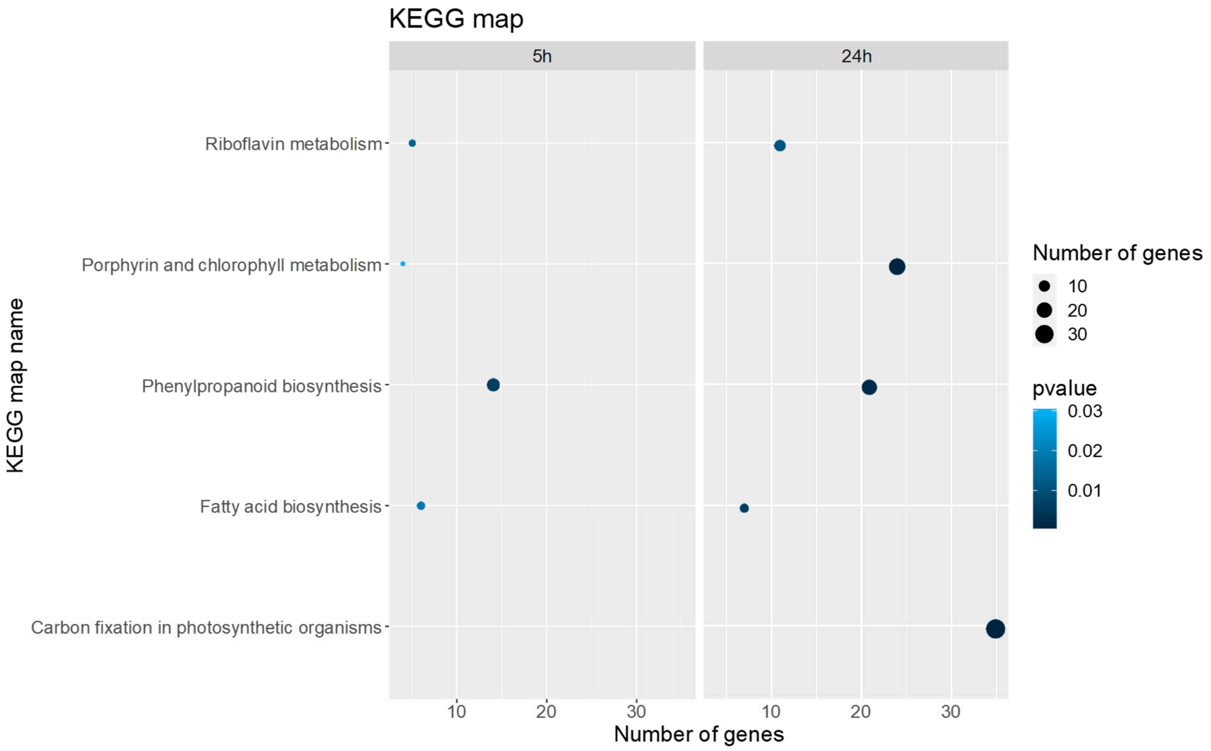

Supplementary Data S8: Medium composition for the NFT tomato culture

A- Final concentrations of the pure salts composing the medium

Pure salts	$Ca(NO_3)_2$	KH_2PO_4	K_2SO_4	$MgSO_4$	$CaSO_4$	EDTA-Fe	Micro-elements (*)
Concentration (mM)	0.5	1.0	1.0	1.5	3.0	0.043	0.3 $mL.L^{-1}$

The nitrogen concentration was set at 1mM which was shown non-limiting for the tomato plant growth (Adamowicz et al., 2008). This concentration was measured and corrected hourly through the automatic system Totomatix with a solution composed by : KNO_3 411mM ; $Ca(NO_3)_2$ 206mM ; $Mg(NO_3)_2$ 88.5mM. (*) see table B for the composition of the micro-element solution.

B- Elemental composition of the micro-element solution

Micro-elements composition	Mo	Mn	Zn	Cu	B	Fe
Concentration (mM)	0.94	38.8	10.8	1.6	68.7	35

Supplementary Data S9: Analytic workflow parameters in Compound Discoverer 3.3

Search description: Untargeted Metabolomics workflow: Find and identify the differences between samples.
- Performs retention time alignment, unknown compound detection, and compound grouping across all samples. Predicts elemental compositions for all compounds, fills gaps across all samples, and hides chemical background (using Blank samples). Identifies compounds using mzVault spectral library (ddMS2) and local compound databases (exact mass or formula). Applies QC-based batch normalization if QC samples are available. Calculates differential analysis (t-test or ANOVA), determines p-values, adjusted p-values, ratios, fold change, CV, etc.). Maps compounds to biological pathways using Metabolika.

Search date: 21/01/2022 17:59:59
Created with Discoverer version: 3.3.0.550
[Input Files (6)]
-->Select Spectra (33)
[Select Spectra (33)]
-->Align Retention Times (ChromAlign) (46)
[Align Retention Times (ChromAlign) (46)]
-->Detect Compounds (49)
[Detect Compounds (49)]
-->Group Compounds (31)
[Group Compounds (31)]
-->Assign Compound Annotations (25)
-->Search mzVault (47)
-->Search Mass Lists (48)
-->Search mzCloud (51)
-->Search ChemSpider (52)
-->Predict Compositions (40)
-->Fill Gaps (32)
[Fill Gaps (32)]
-->Apply SERRF QC Correction (45)
[Assign Compound Annotations (25)]
-->Generate Molecular Networks (53)
[Apply SERRF QC Correction (45)]
-->Mark Background Compounds (28)
[Generate Molecular Networks (53)]
[Map to Metabolika Pathways (43)]
[Search mzVault (47)]
[Search Mass Lists (48)]
[Search mzCloud (51)]
[Search ChemSpider (52)]
[Predict Compositions (40)]
[Mark Background Compounds (28)]
[Differential Analysis (17)]
[Result Exporter (50)]

Processing node 6: Input Files

Processing node 33: Select Spectra

----

1. Spectrum Properties Filter:
- Lower RT Limit: 0
- Upper RT Limit: 0
- First Scan: 0
- Last Scan: 0
- Ignore Specified Scans: (not specified)
- Lowest Charge State: 0
- Highest Charge State: 0
- Min. Precursor Mass: 0 Da
- Max. Precursor Mass: 5000 Da
- Total Intensity Threshold: 0
- Minimum Peak Count: 1
2. Scan Event Filters:
- Mass Analyzer: (not specified)
- MS Order: Any
- Activation Type: (not specified)
- Min. Collision Energy: 0
- Max. Collision Energy: 1000
- Scan Type: Any
- Polarity Mode: (not specified)
- MS1 Mass Range: (not specified)
- FAIMS CV: (not specified)
3. Peak Filters:
- S/N Threshold (FT-only): 1.5
4. Replacements for Unrecognized Properties:
- Unrecognized Charge Replacements: 1
- Unrecognized Mass Analyzer Replacements: ITMS
- Unrecognized MS Order Replacements: MS2
- Unrecognized Activation Type Replacements: CID
- Unrecognized Polarity Replacements: +
- Unrecognized MS Resolution@200 Replacements: 60000
- Unrecognized MSn Resolution@200 Replacements: 30000
5. General Settings:
- Precursor Selection: Use MS(n - 1) Precursor
- Use Isotope Pattern in Precursor Reevaluation: True
- Provide Profile Spectra: Automatic
- Store Chromatograms: False

Processing node 46: Align Retention Times (ChromAlign)
----
1. General Settings:
- Reference File: (not specified)

Processing node 49: Detect Compounds
----
1. General Settings:
- Mass Tolerance [ppm]: 5 ppm
- Min. Peak Intensity: 50000
- Min. # Scans per Peak: 6
- Use Most Intense Isotope Only: True
2. Trace Detection:
- Max. Number of Gaps to Correct: 2

- Min. Number of Adjacent Non-Zeros: 2
3. Peak Detection:
- Chromatographic S/N Threshold: 1.5
- Remove Baseline: False
- Gap Ratio Threshold: 0.35
- Max. Peak Width [min]: 1
- Min. Relative Valley Depth: 0.1
4. Isotope Pattern Detection:
- Group Isotopes for: Br; Cl
- Use Peak Quality for Isotope Grouping: True
- Filter out Features with Bad Peaks Only: True
- Zig-Zag Index Threshold: 0.2
- Jaggedness Threshold: 0.4
- Modality Threshold: 0.9
- Remove Potentially False Positive Isotopes: True
5. Compound Detection:
- Ions:
    [2M+ACN+H]+1
    [2M+ACN+Na]+1
    [2M+FA-H]-1
    [2M+H]+1
    [2M+K]+1
    [2M+Na]+1
    [2M+NH4]+1
    [2M-H]-1
    [2M-H+HAc]-1
    [M+2H]+2
    [M+3H]+3
    [M+ACN+2H]+2
    [M+ACN+H]+1
    [M+ACN+Na]+1
    [M+Cl]-1
    [M+DMSO+H]+1
    [M+FA-H]-1
    [M+H]+1
    [M+H+K]+2
    [M+H+MeOH]+1
    [M+H+Na]+2
    [M+H+NH4]+2
    [M+H-H2O]+1
    [M+H-NH3]+1
    [M+K]+1
    [M+Na]+1
    [M+NH4]+1
    [M-2H]-2
    [M-2H+K]-1
    [M-H]-1
    [M-H+HAc]-1
    [M-H+TFA]-1
    [M-H-H2O]-1
- Base Ions: [M+H]+1; [M-H]-1
- Remove Singlets: True

6. AcquireX Settings:
- Detect Persistent Background Ions: False

Processing node 31: Group Compounds
----
1. General Settings:
- Mass Tolerance: 5 ppm
- RT Tolerance [min]: 0.2
- Align Peaks: False
- Preferred Ions: [M+H]+1; [M-H]-1
- Area Integration: Most Common Ion
2. Peak Rating Contributions:
- Area Contribution: 3
- CV Contribution: 6
- FWHM to Base Contribution: 5
- Jaggedness Contribution: 5
- Modality Contribution: 5
- Zig-Zag Index Contribution: 5
3. Peak Rating Filter:
- Peak Rating Threshold: 0
- Number of Files: 0

Processing node 25: Assign Compound Annotations
----
1. General Settings:
- Mass Tolerance: 5 ppm
2. Data Sources:
- Data Source #1: Predicted Compositions
- Data Source #2: mzCloud Search
- Data Source #3: mzVault Search
- Data Source #4: MassList Search
- Data Source #5: ChemSpider Search
- Data Source #6: Metabolika Search
- Data Source #7: (not specified)
3. Scoring Rules:
- Use mzLogic: True
- Use Spectral Distance: True
- SFit Threshold: 20
- SFit Range: 20
4. Reprocessing:
- Clear Names: False

Processing node 53: Generate Molecular Networks
----
1. Spectral Similarity:
- Use Full MSn Tree: True
- Match Mass Shift: True
- Match Transformations: True
- Variate Transformations: False
- S/N Threshold: 3
- Mass Tolerance: 2.5 mmu
- Min. Fragment m/z: 50

2. Transformations:
- Phase I: (not specified)
- Phase II: (not specified)
- Others: (not specified)
- Max. # Phase II: 1
- Max. # All Steps: 3
3. Applied View Filters:
- Require Transformation: True
- Require MSn: True
- Min. MSn Score: 50
- Min. MSn Coverage: 70
- Min. Fragments: 3
4. Applied Thresholds:
- Require Transformation: False
- Require MSn: False
- Min. MSn Score: 20
- Min. MSn Coverage: 20
- Min. Fragments: 0

Processing node 47: Search mzVault
----
1. Search Settings:
- mzVault Library: Bamba lab 34 lipid mediators library stepped NCE 10 30 45.db|Bamba lab 598 polar metabolites stepped NCE 10 30 45.db|LipidBlast-VS68-Pos.db|Custom mzVault Library.db|Mona libraries_massbank respect etc.db|MSMS-Public-Pos-VS11.db|
- Max. # Results: 10
- Match Factor Threshold: 50
- Search Algorithm: HighChem HighRes
- Match Analyzer Type: False
- IT Fragment Mass Tolerance: 0.4 Da
- FT Fragment Mass Tolerance: 10 ppm
- Use Retention Time: False
- Precursor Mass Tolerance: 10 ppm
- Apply Intensity Threshold: False
- Match Ionization Method: False
- Ion Activation Energy Tolerance: 20
- Match Ion Activation Energy: Any
- Match Ion Activation Type: False
- Compound Classes: All
- Remove Precursor Ion: False
- RT Tolerance [min]: 2

Processing node 48: Search Mass Lists
----
1. Search Settings:
- Mass Lists: Arita Lab 6549 Flavonoid Structure Database.masslist|EFS HRAM Compound Database.masslist|Endogenous Metabolites database 4400 compounds.masslist|Extractables and Leachables HRAM Compound Database.masslist|LipidMaps Structure Database 2021-09-13.massList|Natural Products Atlas 2020_06.masslist|
- Mass Tolerance: 5 ppm
- Use Retention Time: False
- RT Tolerance [min]: 2

Processing node 51: Search mzCloud
----
1. General Settings:
- Compound Classes: All
- Precursor Mass Tolerance: 10 ppm
- FT Fragment Mass Tolerance: 10 ppm
- IT Fragment Mass Tolerance: 0.4 Da
- Library: Autoprocessed; Reference
- Post Processing: Recalibrated
- Max. # Results: 10
- Annotate Matching Fragments: False
- Search MSn Tree: False
2. DDA Search:
- Identity Search: HighChem HighRes
- Match Activation Type: True
- Match Activation Energy: Match with Tolerance
- Activation Energy Tolerance: 20
- Apply Intensity Threshold: True
- Similarity Search: None
- Match Factor Threshold: 60
3. DIA Search:
- Use DIA Scans for Search: False
- Max. Isolation Width [Da]: 500
- Match Activation Type: False
- Match Activation Energy: Any
- Activation Energy Tolerance: 100
- Apply Intensity Threshold: False
- Match Factor Threshold: 20

Processing node 52: Search ChemSpider
----
1. Search Settings:
- Database(s):
    AraCyc
    Aurora Fine Chemicals
    Baoji Herbest Bio-Tech
    Extrasynthese
    FooDB
    Golm Metabolome Database
    Human Metabolome Database
    Indofine
    LipidMAPS
    MassBank
    Phenol-Explorer
    PlantCyc
    Sequoia Research Products
- Search Mode: By Formula or Mass
- Mass Tolerance: 5 ppm
- Max. # of results per compound: 100
- Max. # of Predicted Compositions to be searched per Compound: 3
- Result Order (for Max. # of results per compound): Order By Reference Count (DESC)

2. Predicted Composition Annotation:
- Check All Predicted Compositions: False

Processing node 40: Predict Compositions
----
1. Prediction Settings:
- Mass Tolerance: 5 ppm
- Min. Element Counts: C H
- Max. Element Counts: C90 H190 Br3 Cl4 N10 O18 P3 S5
- Min. RDBE: 0
- Max. RDBE: 40
- Min. H/C: 0.1
- Max. H/C: 3.5
- Max. # Candidates: 10
- Max. # Internal Candidates: 200
2. Pattern Matching:
- Intensity Tolerance [%]: 30
- Intensity Threshold [%]: 0.1
- S/N Threshold: 3
- Min. Spectral Fit [%]: 30
- Min. Pattern Cov. [%]: 90
- Use Dynamic Recalibration: True
3. Fragments Matching:
- Use Fragments Matching: False
- Mass Tolerance: 5 ppm
- S/N Threshold: 3

Processing node 32: Fill Gaps
----
1. General Settings:
- Mass Tolerance: 5 ppm
- S/N Threshold: 1.5
- Use Real Peak Detection: True

Processing node 45: Apply SERRF QC Correction
----
1. General Settings:
- Min. QC Coverage [%]: 66
- Max. QC Area RSD [%]: 30
- Max. Corrected QC Area RSD [%]: 25
- Max. # Files Between QC Files: 25
- Correct Blank Files: False
- # Batches: 1
- Interpolate Gap-filled QC Areas: True
2. Random Forest Settings:
- # Trees: 200

Processing node 28: Mark Background Compounds
----
1. General Settings:
- Max. Sample/Blank: 5
- Max. Blank/Sample: 0

- Hide Background: True

Processing node 17: Differential Analysis
----
1. General Settings:
- Log10 Transform Values: True
2. Peak Rating Contributions:
- Update Peak Rating: True
- Area Contribution: 3
- CV Contribution: 10
- FWHM to Base Contribution: 5
- Jaggedness Contribution: 5
- Modality Contribution: 5
- Zig-Zag Index Contribution: 5

Processing node 50: Result Exporter
----
1. Output Data:
- Export all Columns: False
- File Name: (not specified)
- Export Format: Text (tab-delimited) (.txt)
2. Text Export Options:
- R-friendly Headers: True

Supplementary Data S10 : List of genes and primers

Genes	Gene annotation	Metabolic pathway	Forward primer	Reverse primer	Application
Solyc01g091160	Arginase	Polyamine	AGGTTCGGTGTGGAGCAATA	CCACATCAGCACCAACAACA	qPCR
Solyc01g091170	Arginase	Polyamine	TTTTGAGTCAGGCGGTCTCT	TTGGACATCTTGGCAGCAAG	qPCR
Solyc01g110440	ADC	Polyamine	CGGCCTGTAATTGGACTTCG	AGCAAGCAACTCTGTTGTGG	qPCR
Solyc04g082030	ODC	Polyamine	CCATTAGCGTGCATGTCGAA	AGGTGAGTAACAATGGCGGA	qPCR
Solyc03g007240	SPMS	Polyamine	TTTAAGGAGGGAGGTGAGCG	CTATGCGTGTGACAAGTGGG	qPCR
Solyc03g117600	HCT	Phenylpropanoid	TATGAGATGTTGGCAGGGCA	ATCACCAGCAACAGCAACAG	qPCR
Solyc06g068650	4CL	Phenylpropanoid	ATGATCCAGAGGCCACTACG	CTCCTGCTTGCTCGTCTTTC	qPCR
Solyc09g007910	PAL5	Phenylpropanoid	TTTCTCCATTACAAATCAAACCA	TTCACTTCATCCAAATGACTCC	qPCR
Solyc11g071480	HCT_like PHT	Phenylpropanoid	TATCGAGAGTGGGCAGGAAG	TCCAATGAGGTGTCCACAGA	qPCR
Solyc11g071470	HCT_like PHT	Phenylpropanoid	CTGTAGACGCCTCGTTGGAC	AGGAGAGCATAGAGGGAGAAGG	qPCR
Solyc08g006960	CAC	House-keeping gene	CCTCCGTTGTGATGTAACTGG	ATTGGTGGAAAGTAACATCATCG	qPCR
Solyc10g049850	TIP41_like protein	House-keeping gene	ATGGAGTTTTTGAGTCTTCTGC	GCTGCGTTTCTGGCTTAGG	qPCR
Solyc11g071470	HCT_like PHT	Phenylpropanoid	ACAACGGATCCATGAATGTGAA AATTGAGAGTTCAAAAATC	ACAAGCGGCCGCTCACTTTGCTTTC AAATCTA	cloning
Solyc11g071480	HCT_like PHT	Phenylpropanoid	ACAACGGATCCATGAATGTGAA AATTGATAGTTCAAAAATC	ACAAGCGGCCGCTCACTTTGCTTTC AAATCTA	cloning

ADC: Arginine decarboxylase ; ODC: Ornithine decarboxylase ; SPMS : Spermine synthase ; HCT: Hydroxycinnamoyl quinate/shikimate hydroxycinnamoyl transferase ; 4CL: 4-coumarate CoEnzyme A ligase ; PAL: Phenylalanine ammonia lyase ; PHT: Putrescine hydroxycinnamoyl transferase ; CAC: Clathrin adaptor complexe ; TIP41: TAP42 interacting protein of 41kDa.
